# Evaluating the fitness consequences of plasticity in tolerance to pesticides

**DOI:** 10.1002/ece3.6211

**Published:** 2020-04-12

**Authors:** Devin G. DiGiacopo, Jessica Hua

**Affiliations:** ^1^ Biological Sciences Department Binghamton University (SUNY) Binghamton New York

**Keywords:** carbaryl, insecticide, *Lithobates sylvaticus*, trade‐offs

## Abstract

In a rapidly changing world, phenotypic plasticity may be a critical mechanism allowing populations to rapidly acclimate when faced with novel anthropogenic stressors. Theory predicts that if exposure to anthropogenic stress is heterogeneous, plasticity should be maintained as it allows organisms to avoid unnecessary expression of costly traits (i.e., phenotypic costs) when stressors are absent. Conversely, if exposure to stressors becomes constant, costs or limits of plasticity may lead to evolutionary trait canalization (i.e., genetic assimilation). While these concepts are well‐established in theory, few studies have examined whether these factors explain patterns of plasticity in natural populations facing anthropogenic stress. Using wild populations of wood frogs that vary in plasticity in tolerance to pesticides, the goal of this study was to evaluate the environmental conditions under which plasticity is expected to be advantageous or detrimental. We found that when pesticides were absent, more plastic populations exhibited lower pesticide tolerance and were more fit than less plastic populations, likely avoiding the cost of expressing high tolerance when it was not necessary. Contrary to our predictions, when pesticides were present, more plastic populations were as fit as less plastic populations, showing no signs of costs or limits of plasticity. Amidst unprecedented global change, understanding the factors shaping the evolution of plasticity will become increasingly important.

## Graphical abstract

Wood frog (*Lithobates sylvaticus*) adult, Elk State Forest, PA. Populations of wood frogs provided a useful tool to investigate the environmental factors promoting the maintenance or loss of phenotypic plasticity. Image Credit: Devin DiGiacopo.

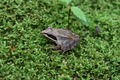

## 1 INTRODUCTION

Global expansion and intensification of anthropogenic impacts have created innumerable challenges for wildlife populations (Aronson et al., 2014; Ellis & Ramankutty, 2008). Organisms faced with these new challenges must escape, acclimate, adapt, or perish (Beketov, Kefford, Schäfer, & Liess, 2013; Parmesan, 2006). If change is rapid, adaptation via selection on standing genetic variation might not act quickly enough to prevent population decline or collapse (Bürger & Lynch, 1995). On the other hand, phenotypic plasticity (i.e., the ability of a single genotype to produce multiple phenotypes depending on its environment) creates potential for rapid acclimation to these novel conditions within a single generation. Across longer time scales, phenotypic plasticity may even promote evolutionary innovation and adaptation by exposing otherwise cryptic genetic variation to selection (Diamond & Martin, 2016). For these reasons, plasticity is likely an essential mechanism allowing natural populations to persist when faced with anthropogenic change (Scheiner, Barfield, & Holt, 2019; Snell‐Rood, Kobiela, Sikkink, & Shephard, 2018). However, plasticity is not omnipresent; while plasticity may allow populations to persist initially in the presence of novel stressors, subsequent trait canalization may occur (i.e., via genetic assimilation; Waddington, 1953) due to costs or limits of plastic expression (Dewitt, Sih, & Wilson, 1998). Examining the implications of anthropogenic change for the maintenance of or selection against plasticity is crucial to understanding the evolutionary consequences of human activities for wild populations.

In variable environments, where the fitness optimum for a given trait remains in flux, plasticity is theorized to be favored (Lyytinen, Brakefield, Lindström, & Mappes, 2004). A primary benefit of plasticity under these circumstances is that it allows organisms to avoid phenotypic costs (i.e., costs of expressing a suboptimal or incorrect phenotype for a particular environment; Auld, Agrawal, & Relyea, 2010; Callahan, Maughan, & Steiner, 2008; Murren et al., 2015). Phenotypic costs may arise when organisms express traits that are adaptive in the presence of anthropogenic stressors, but costly when these stressors are absent. For example, larval anurans with relatively high tolerance to an insecticide had lower survival rates than their half‐siblings with lower tolerance in insecticide‐free environments (Semlitsch, Bridges, & Welch, 2000). Likewise, in a weedy plant species, genotypes with high tolerance to an herbicide produced fewer seeds than relatively susceptible genotypes in herbicide‐free environments (Baucom & Mauricio, 2004). These studies suggest tolerance to contaminants is costly and may impose a fitness decrement when contaminants are absent. Therefore, as humans introduce new, often heterogeneously occurring stressors, avoiding phenotypic costs may be one mechanism that promotes the maintenance of phenotypic plasticity.

Conversely, if environmental conditions stabilize (e.g., exposure to a contaminant becomes persistent), costs or limitations associated with plastic expression may lead to trait canalization through the process of genetic assimilation (Pigliucci, 2005). For example, costs of plasticity may arise due to the maintenance of molecular or physiological machinery needed to accurately detect the environment and produce a suitable phenotype (Dewitt et al., 1998; Van Kleunen & Fischer, 2005). Costs of plasticity are measured as the reduction in fitness exhibited by a more plastic genotype compared to a less plastic genotype when expressing the same phenotype (Callahan et al., 2008). For example, Agrawal, Conner, Johnson, and Wallsgrove (2002) measured concentrations of glucosinolates (a class of antiherbivore compounds) in wild radishes and found that half‐sibling families exhibiting greater plasticity in glucosinolate production had lower fitness than less plastic families in herbivore‐free environments, despite expressing similar glucosinolate concentrations. Costs of plasticity may therefore promote canalization of previously plastic traits via genetic assimilation, especially in instances where exposure to anthropogenic stressors becomes homogenous.

In addition to costs, fixed expression may be favored over plastic expression due to limits of plasticity. Limits of plasticity arise when, in a given environment, highly plastic genotypes cannot produce a trait value as close to the fitness optimum as less plastic genotypes. Because the environment dictates the fitness optimum, limits are measured as the difference in trait values produced by genotypes exhibiting varying degrees of plasticity, *within* a given environment (Dewitt et al., 1998; Murren et al., 2015). Limits of plasticity may occur due to a variety of reasons, including unreliable environmental cues, the lag between cue detection and phenotype production, the inability to produce extreme phenotypes, the relative inefficacy of a trait produced later in development compared to earlier in development, and antagonistic interactions between different plastic responses and environmental factors across time (Cipollini, 2004; Dewitt et al., 1998; Valladares, Gianoli, & Gómez, 2007; Weinig & Delph, 2001). However, to date, there is little evidence for certain types of limits, such as the inability of highly plastic genotypes to produce extreme phenotypes (i.e., developmental constraints; Auld et al., 2010; Dewitt et al., 1998; Kleunen, Fischer, & Schmid, 2000; Lind & Johansson, 2009). Therefore, while limits of plasticity may promote genetic assimilation, it remains unknown whether such limits exist in traits involved in responses to anthropogenic change.

Despite the number of theoretical studies exploring the factors promoting the maintenance or loss of plasticity (i.e., genetic assimilation), few have applied these concepts to wild populations (Pigliucci & Murren, 2003), especially those facing anthropogenic change. With increasing demands on agriculture and the encroachment of industry and urbanization on natural areas, pesticide contamination in natural ecosystems offers us the opportunity to evaluate this gap. Wildlife populations across a diversity of taxa have evolved increased tolerance to pesticides (Coors, Vanoverbeke, Bie, & Meester, 2009; Cothran, Brown, & Relyea, 2013; Roush & Tabashnik, 2012; Scott & Kasai, 2004). In some populations, tolerance can be increased when pesticides are present in the environment (i.e., via plasticity), whereas in others, tolerance is not influenced by the presence of pesticides (i.e., fixed or constitutive expression; Hua, Morehouse, & Relyea, 2013). Recent work on wild populations of wood frogs (*Lithobates sylvaticus*) demonstrated that landscape patterns of plasticity in pesticide tolerance are consistent with the process of genetic assimilation (Hua et al., 2015). In this study, the authors hypothesized that tolerance to the insecticide carbaryl was an ancestrally plastic trait and was evolutionary canalized over time as populations increasingly faced persistent exposure to the chemical. Indeed, using a space for time substitution approach, they found that populations far from agriculture (proxy for ancestral populations) which are infrequently, if ever, exposed to pesticides, expressed low tolerance in the absence of pesticides, but could increase tolerance in the presence of pesticides (i.e., high plasticity in tolerance). In contrast, populations of wood frogs living close to agriculture (proxy for derived populations), where exposure to pesticides is likely more frequent and intense, typically expressed high tolerance whether pesticides were present or absent (i.e., low plasticity in tolerance). These populations provide a unique opportunity for investigating factors (i.e., benefits or costs) that might promote the maintenance or loss of phenotypic plasticity in natural populations facing anthropogenic change.

Therefore, we chose four wood frog populations from Hua et al. (2015) across a spatial gradient (distance to agriculture) as a proxy for populations at different points in time during the process of genetic assimilation, such that populations far from agriculture represented ancestral populations (higher plasticity in tolerance) and populations near agriculture represented derived populations (lower plasticity in tolerance). The goal of this study was to evaluate the environmental circumstances under which plasticity is advantageous or detrimental. Specifically, in pesticide‐free environments (representative of the ancestral environment), plasticity should be advantageous if it allows organisms to avoid phenotypic costs associated with expressing high tolerance when it is not necessary. Conversely, in pesticide environments (representative of derived environments), plasticity may be detrimental due to costs or limits of plastic expression. Therefore, we tested two predictions: (a) In pesticide environments, more plastic populations will be less fit than less plastic populations, while (b) in pesticide‐free environments, more plastic populations will be more fit than less plastic populations.

## 2 MATERIALS AND METHODS

### 2.1 Model pesticide

In this study, we used carbaryl (Sevin© commercial formulation; 22.5% active ingredient; CAS#: 63‐25‐2) as our model pesticide because previous studies have demonstrated that populations of wood frogs exhibit varying levels of plasticity in tolerance to carbaryl (Hua et al., 2015). Carbaryl is an acetylcholinesterase (AChE)‐inhibiting carbamate insecticide, used in homes, gardens, and agriculture (Atwood & Paisley‐Jones, 2017). Carbaryl can be detected in freshwater systems at concentrations ≤1.5 mg/L (Norris, Lorz, & Gregory, 1983; Peterson et al., 1994). The sublethal concentrations used in this study have been shown to cause an increase in tolerance in plastic populations of wood frogs, without causing acute toxicity (Hua et al., 2013, 2015).

### 2.2 Animal collection and husbandry

To test our predictions, we identified four wood frog populations along the ancestral to derived gradient (ranging from low to high plasticity in tolerance to pesticides accordingly) established by Hua et al., 2015. On 12 March 2016, we collected 10 partial egg masses from each of 4 wood frog populations in western Pennsylvania, (“SQR,” “BJ,” “HOP,” and “TRL”; Hua et al., 2015). Embryos were transported to Binghamton University's Ecological Research Facility (ERF) and reared in 100‐L pools, filled with 90 L of well water and covered in 70% shade cloth, until hatching. On 27 March, we moved a randomly selected subset of hatchlings from each population to the laboratory, where they were held in 17‐L Sterilite© bins containing 10 L aged well water at equal densities (35 tadpoles per bin) until the start of the experiments.

### 2.3 Time‐to‐death experiment

**FIGURE 1:**
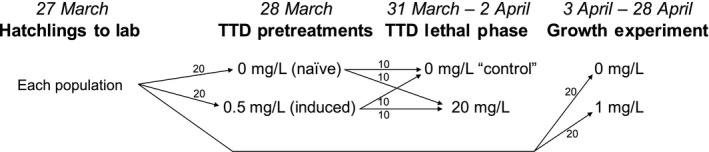
Schematic illustrating experimental design and distribution of wood frog larvae to TTD and growth experiments. All four populations followed the same design

To quantify the degree of plasticity exhibited by individuals in each of the four populations, we conducted a time‐to‐death assay. On 28 March, we haphazardly placed 25 hatchlings from each population into 17‐L Sterilite© bins containing 10 L of 0.5 mg/L carbaryl solution (“sublethal pesticide pretreatment”) or 10 L of pesticide‐free aged well water (“pesticide‐free pretreatment”; Figure 1). Hatchlings remained in their pretreatments for 72 hr. Pesticides were not renewed and hatchlings were not fed because they were still receiving nutrients from their yolk sacs. On 31 March, once hatchlings reached the tadpole stage (Gosner stage 25; Gosner, 1960), we began the TTD assay. We placed 10 tadpoles from each pretreatment into individual 3‐oz plastic cups, containing 50 ml of either a 20 mg/L (lethal) carbaryl solution, or a 0 mg/L (pesticide‐free) control, for a total of 160 units (Figure 1). All individuals in lethal pesticide treatments died by hour 44 (2 April). All individuals in pesticide‐free controls survived to the end of the experiment.

#### 2.3.1 Statistical analyses

**FIGURE 2:**
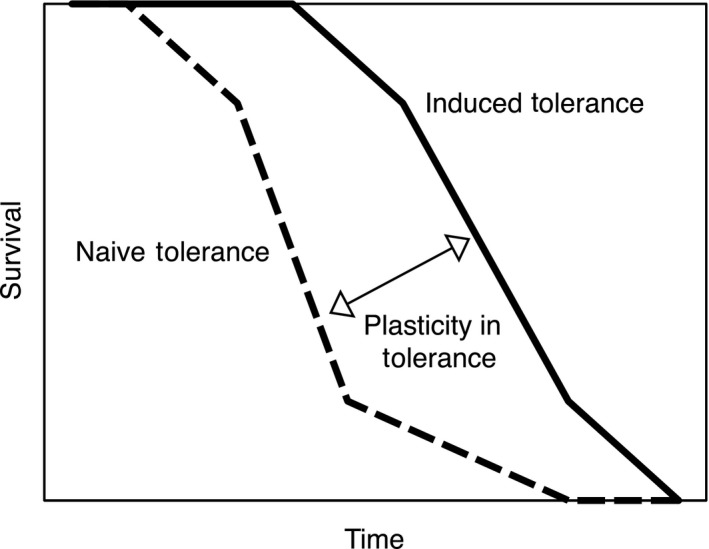
Example survival curves representing naïve tolerance (dashed line), induced tolerance (solid line), and plasticity (arrow between lines). Naïve tolerance is measured as a population's time to death in a lethal dose of carbaryl after pesticide‐free pretreatments. Induced tolerance is measured as a population's time to death in a lethal dose of carbaryl after sublethal pesticide pretreatments. Plasticity is a measure of the difference in naïve and induced tolerance for a population

Using Wilcoxon–Gehan survival analyses, we compared the survival of tadpoles exposed to lethal concentrations of carbaryl for each population and both pretreatments. These analyses compare differences in survival over time for one or more factors (Altman, Machin, Bryant, & Gardner, 2013). This allowed us to quantify tolerance (i.e., time to death in a lethal concentration) exhibited by a population after each pretreatment (pesticide‐free or sublethal pesticide). We designate “naïve tolerance” as time to death (in a lethal dose of carbaryl) after pesticide‐free pretreatments and “induced tolerance” as time to death following sublethal pesticide pretreatments (Figure 2). Then, to assess plasticity in tolerance for each population we used pairwise comparisons of time to death for individuals from pesticide‐free pretreatments (naïve tolerance) to that of individuals reared in sublethal pesticide pretreatments (induced tolerance). Populations with a greater difference between naïve and induced tolerance exhibit higher plasticity in pesticide tolerance (represented by a larger Wilcoxon–Gehan statistic).

### 2.4 Growth experiment

To test our predictions regarding the environment‐specific advantages or disadvantages of plasticity in tolerance, we examined tadpole mass, snout–vent length and development in pesticide‐free and sublethal pesticide environments. Prior to the start of this experiment (31 March–3 April), we fed tadpoles ground Tetramin© fish flakes ad libitum. On 3 April, we divided 40 randomly selected tadpoles from each population into individual experimental units representing two treatments—2‐L deli cups filled with 1 L of either 0 mg/L (“pesticide‐free environment”) or 1 mg/L (“sublethal pesticide environment”) carbaryl solution—for a total of 160 experimental units (Figure 1). As limited resources are a fundamental requirement of resource allocation experiments, we reared all tadpoles on a fixed diet of 2% of their body weight per day (Hammond, Jones, Stephens, & Relyea, 2012). This was calculated as the average body weight of three extra (not used for TTD or growth experiments) individuals from each population. At the start of the experiment (3 April), average mass across the four populations was 0.033 ± 0.011 g (mass ± standard error), meaning each tadpole received 0.0007 g food per day from 3 April through 18 April. On 18 April, average mass was measured again following the same procedure. The new average was 0.046 g ± 0.009 g, making the ration 0.0009 g daily, from 18 April through the end of the experiment on 28 April (Table 1).

**TABLE 1:** Average mass ± standard error (grams) of extra tadpoles used to determine food rations (top two rows) and total mortality of individuals in the growth experiment (bottom two rows)

Mass (g)	BJ	HOP	TRL	SQR	Total
3 April	0.025 ± 0.006	0.029 ± 0.002	0.035 ± 0.004	0.047 ± 0.003	0.033 ± 0.011
18 April	0.037 ± 0.006	0.055 ± 0.004	0.051 ± 0.001	0.043 ± 0.002	0.046 ± 0.009

Mortality	BJ	HOP	TRL	SQR	Total
Pesticide‐free	1	4	5	0	10
Sublethal pesticide	3	1	2	0	6

Overall survival for the duration of the growth experiment was 90% (Table 1). Two tadpoles died within the first 48 hr of the experiment (by 5 April) and were replaced using extra tadpoles from population‐specific bins held under the same conditions. Fourteen tadpoles died within the last 24 days of the experiment and were not replaced. Every five days, we conducted water changes and renewed pesticide solutions for each pesticide treatment unit. On 28 April, all surviving tadpoles were euthanized using an overdose of MS‐222 and preserved individually in a 10% formalin solution. We then measured the mass, snout–vent length (SVL), and Gosner stage (Gosner, 1960) of each tadpole. These metrics are commonly regarded as proxies for amphibian fitness, with larger larvae typically being less susceptible to predation, achieving a larger size at metamorphosis, reaching sexual maturity earlier, and having higher reproductive outputs (Pereira & Maneyro, 2012; Semlitsch, Scott, & Pechmann, 1988; Smith, 1987; Werner, 1986).

#### 2.4.1 Statistical analyses

While we measured mass, SVL, and Gosner stage, all metrics were highly correlated (*p* < .001). Therefore, we focused on mass to test our predictions because studies suggest that mass is a more consistent predictor of postmetamorphic performance (Earl & Whiteman, 2015). To assess the relative fitness between populations in pesticide and pesticide‐free environments, we conducted a univariate ANOVA with population and environment (sublethal pesticide versus pesticide‐free) as independent variables. The two replacement individuals (for individuals that died in the first 48 hr) and the five individuals that died within the last 48 hr experienced experimental conditions for at least 24 out of the 26 days of the experiment. Therefore, we included them in the ANOVA for growth comparisons. Nine tadpoles died between 6 April and 25 April. These individuals experienced experimental conditions for <24 out of the 26 days and were subsequently excluded from the ANOVA for growth comparisons. All statistical analyses were conducted in SPSS (version 24, IBM).

## 3 RESULTS

### 3.1 Naïve tolerance

**FIGURE 3:**
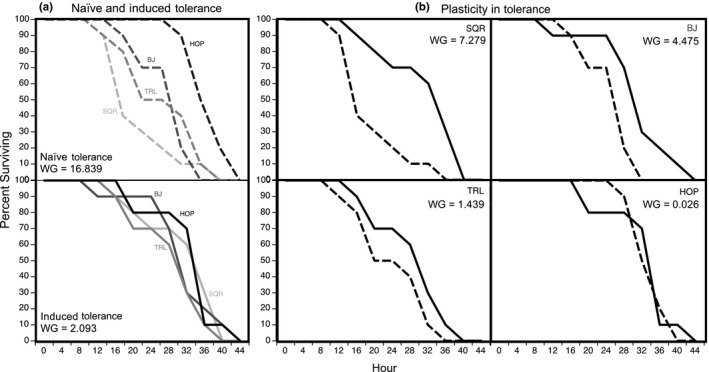
(a) Survivorship curves representing population variation in naïve tolerance (top panel; dashed lines) and induced tolerance (bottom panel; solid lines). Wilcoxon–Gehan statistics (“WG”) compare survival curves among populations (a greater value represents greater variation in survival). (b) Each panel illustrates the naïve (dashed lines) and induced (solid lines) tolerance for a single population. A large Wilcoxon–Gehan statistic represents a large difference between naïve and induced tolerance and therefore, high plasticity in tolerance

Wilcoxon's survival analyses demonstrated that there was significant variation in naïve tolerance among populations (Wilcoxon–Gehan = 16.839, *p* = .001; Figure 3a, top panel). HOP exhibited higher naïve tolerance than TRL (Wilcoxon–Gehan = 6.984; *p* = .008), BJ (Wilcoxon–Gehan = 10.438; *p* = .001), and SQR (Wilcoxon–Gehan = 10.910; *p* = .001). BJ had higher naïve tolerance than SQR (Wilcoxon–Gehan = 4.415; *p* = .036) but not TRL (Wilcoxon–Gehan = 0.006; *p* = .938). TRL and SQR exhibited similar levels of naïve tolerance (Wilcoxon–Gehan = 2.035; *p* = .154; Figure 3a, top panel).

### 3.2 Induced tolerance

All populations exhibited similar levels of induced tolerance (Wilcoxon–Gehan = 2.093, *p* = .553; Figure 3a, bottom panel).

### 3.3 Plasticity in tolerance

Wilcoxon's survival analyses indicated that tadpoles from SQR (Wilcoxon–Gehan = 7.279; *p* = .938) demonstrated the highest degree of plasticity in tolerance, followed by BJ (Wilcoxon–Gehan = 4.475; *p* = .034), TRL (Wilcoxon–Gehan = 1.439; *p* = .230), and HOP (Wilcoxon–Gehan = 0.026; *p* = .873; Figure 3b).

### 3.4 Fitness

**FIGURE 4:**
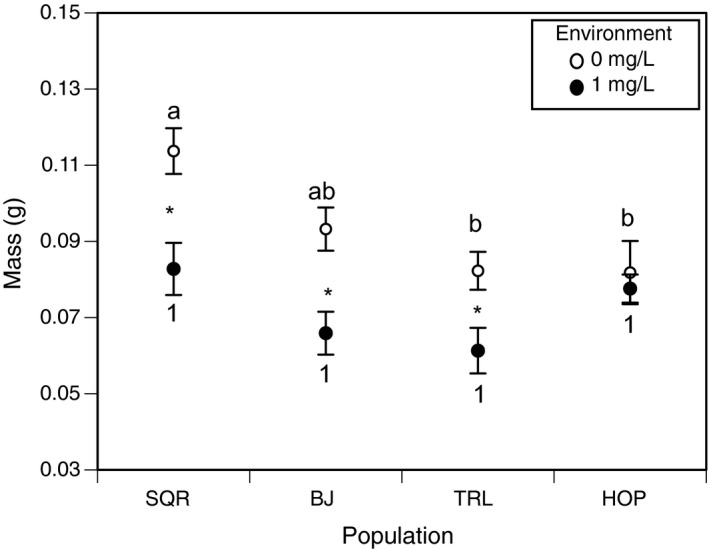
Average mass (in grams) of tadpoles from each population when reared in pesticide‐free (white dots) and sublethal pesticide (black dots) environments. Asterisks denote significant differences in mass across environments *within* populations. Shared letters denote nonsignificant differences among populations in the pesticide‐free environment. Shared numbers denote nonsignificant differences in mass among populations in the sublethal pesticide environment. Populations are arranged in order of decreasing plasticity from left to right on the *x*‐axis

ANOVAs demonstrated a significant effect of our model for mass (*F* = 7.396, *p* < .001). There were significant effects of both population (*F* = 6.711, *p* < .001) and environment (*F* = 23.079, *p* < .001) on tadpole mass. However, there was no interaction between population and environment (*F* = 1.966, *p* = .122) on mass. Despite the absence of a significant interaction, we include Bonferroni‐adjusted pairwise comparisons between populations and treatments, as they are central to testing our predictions (Wei, Carroll, Harden, & Wu, 2012). In pesticide‐free environments, SQR was larger than both TRL and HOP, but not BJ (Figure 4). In sublethal pesticide environments, all populations reached the same size (Figure 4). Individuals from SQR (*p* < .001), BJ (*p* = .002) and TRL (*p* = .025) were significantly larger when reared in pesticide‐free environments than in pesticide environments, while individuals from HOP were not (*p* = .619; Figure 4).

## 4 DISCUSSION

Contrary to our predictions, in pesticide environments, we did not find evidence that more plastic populations were less fit than less plastic populations. In our time‐to‐death assay, we observed that more plastic populations exhibited induced tolerance levels commensurate with those of less plastic populations, suggesting that there were no limits of plasticity in expressing high tolerance when necessary. In our growth experiment, more plastic populations reached the same size as less plastic populations, suggesting that there were no costs of plasticity in pesticide environments. Collectively, these results suggest that under our experimental conditions, plasticity in tolerance was not detrimental in the presence of pesticides. These results do not support the hypothesis proposed in Hua et al. (2015) that costs of plasticity may have promoted genetic assimilation in wood frog populations facing frequent pesticide exposure near agricultural systems. However, costs of plasticity have been notoriously difficult to demonstrate (Van Buskirk & Steiner, 2009) and may be negligible in all but highly plastic genotypes (Lind & Johansson, 2009). Additionally, both costs and limits of plasticity may be diminished in environments with abundant resources (Auld et al., 2010; Moret & Schmid‐Hempel, 2000; Snell‐Rood et al., 2015; Wuerthner et al., 2019). For this reason, we reared our individuals on restricted diets, but cannot be sure that resources did not play a role in the observed absence of costs or limits. Alternatively, there may have been costs and limits of plasticity which we simply did not capture. Carbaryl tolerance is a complex trait involving multiple enzymes, such as acetylcholinesterase, microsomal oxidases, glutathione transferases, hydrolases, and reductases, many of which serve multiple biological purposes (Hua et al., 2013; Qin et al., 2012; Yu, 1992). There may have been limits of plasticity in the production of specific enzymes, or costs of plasticity manifesting in other physiological or morphological features, especially if a broader range of pesticide concentrations were included.

On the other hand, in the absence of pesticides, more plastic populations were generally larger than less plastic populations, supporting our prediction that plasticity would be beneficial when pesticides were not present. This was likely because more plastic populations avoided phenotypic costs of tolerance when it was unnecessary to express tolerance. For instance, SQR exhibited the lowest naïve tolerance and reached the largest size, while HOP exhibited the highest naïve tolerance and reached the smallest size in pesticide‐free environments, suggesting that expenditure of resources on pesticide tolerance may reduce resources available for growth. These results corroborate previous studies demonstrating the presence of phenotypic costs of tolerance, such as reduced fecundity or survival of tolerant individuals in pesticide‐free environments (Baucom & Mauricio, 2004; Semlitsch et al., 2000). Beyond life history trade‐offs (e.g., survival and reproduction), high tolerance to pesticides has been linked with reduced tolerance to biotic stressors, such as parasites (Hua et al., 2017; Jansen, Stoks, Coors, Doorslaer, & Meester, 2011). Additionally, these findings are coherent with observations from Hua et al. (2015), where high plasticity in tolerance was most common in environments where pesticide exposure was likely infrequent or absent (i.e., far from agriculture). Taken together, these results support theoretical predictions that selection may favor plasticity in traits associated with responses to anthropogenic stress when exposure is infrequent.

While this study provides novel insight into the implications of anthropogenic change for selection on plasticity in wild populations, we assert that further research is needed to support the patterns we observed. First, we note an interesting disparity between the plasticity in tolerance exhibited by HOP in our study as compared Hua et al. (2015). In their paper, HOP was among the most plastic populations of all 15 populations sampled, while in our study HOP was the least plastic population. This discrepancy may have been the result of sampling different portions of the population's total genetic diversity, or because of a strong selective event of which we are unaware. Despite this, having quantified plasticity anew for the individuals in our experiment, we believe that this population was still useful in examining the implications of plasticity in tolerance for fitness across environments. Additionally, while the general patterns of fitness we observed support the notion that plasticity is beneficial in pesticide‐free environments (likely due to the avoidance of phenotypic costs), some pairwise comparisons between populations were not statistically significant (Figure 4). Future studies could improve their statistical power or employ other models for addressing similar questions by including more populations. Still, we believe that these results provide important initial insight into the factors promoting the maintenance of plasticity or genetic assimilation in populations facing anthropogenic change.

In considering the fate of such populations, it is important to note that they naturally exist within much more complex ecological systems. We are only beginning to understand the ecological implications of phenotypic plasticity (Nussey, Wilson, & Brommer, 2007), but it is already apparent that plasticity is important in moderating organisms' interactions with their biotic and abiotic environments (Miner, Sultan, Morgan, Padilla, & Relyea, 2005). Conversely, these biotic (e.g., predators, pathogens, and competitors) and abiotic (e.g., temperature, resources, and contaminants) environmental factors may trigger plastic responses that limit future plastic responses (Cipollini, 2004; Weinig & Delph, 2001). These same factors may also impact the availability of resources and resource demands on organisms, potentially exposing costs or imposing further limitations on plasticity (Auld et al., 2010; Valladares et al., 2007). In the same vein, we might expect that plastic responses to anthropogenic change may constrain organisms' capacity for plastic responses to other environmental factors, potentially influencing both their ecology and evolution. However, to date, these indirect, eco‐evolutionary effects of anthropogenic activity remain largely unexplored and represent an important future direction (Brunner, Deere, Egas, Eizaguirre, & Raeymaekers, 2019; Hendry, Gotanda, & Svensson, 2017). Although we examined the fitness implications of plasticity in a highly simplified pair of environments, our findings may serve as a first step in understanding the broader implications of plasticity in traits relevant to anthropogenic change in more ecologically realistic systems. We demonstrate that plasticity allows organisms to acclimate rapidly to multiple environments, seemingly without additional limits or costs in one metric of fitness. Therefore, as natural populations face increasing exposure to anthropogenic stressors, often heterogeneously across time and space, evolution may favor phenotypic plasticity in traits associated with tolerating them.

## CONFLICT OF INTEREST

None declared.

## AUTHOR CONTRIBUTIONS

**Devin G. DiGiacopo:** Conceptualization (lead); data curation (lead); formal analysis (lead); investigation (lead); methodology (lead); project administration (lead); resources (supporting); software (lead); supervision (equal); validation (equal); visualization (lead); writing – original draft (lead); writing – review and editing (supporting). **Jessica Hua:** Conceptualization (supporting); data curation (supporting); formal analysis (supporting); funding acquisition (lead); investigation (supporting); methodology (supporting); project administration (supporting); resources (lead); software (supporting); supervision (equal); validation (equal); visualization (supporting); writing – original draft (supporting); writing – review and editing (lead).

## Data Availability

Data for this study are available at the Dryad Digital Repository: https://doi.org/10.5061/dryad.4xgxd2561
